# Henke’s Atlas of Surgical Anatomy

**Published:** 1884-10

**Authors:** 


Henke's Atlas of Surgical Anatomy.—A series of plates illustrating the application of Anatomy to Medicine and Surgery. Translated and edited by W. A. Rothacker, M.D., Pathologist to Cincinnati Hospital; Lecturer on Pathological Anatomy in Miami Medical College. A. E. Wilde & Co., Publishers. Cincinnati, 1884. Price, $10. N. D. McDonald & Co., Agents, Atlanta, Ga.

This is a work of rare merit, and deserves to be in the library of every physician in the land. The large size of the plates and their anatomical accuracy are to be found in no other work we have seen. We heartily commend it to every one.

We have received from Richard A. Saalfield, 12 Bible House, New York, the following pieces of music:

With Cleveland we shall win the day, by J. P. Skelly.

Cleveland and Hendricks' Grand Victory March, by J. J. Freeman.

You Ask Me to Forgive the Past, by Ed. Green, a very taking little, sentimental ballad, full of melody, which will surely find its way to the hearts of all.

Better Luck To-Morrow, by Henry Martyn. A new motto song; full of hope, good cheer and downright sensible thought. Music very good. Words excellent.

Amatori Waltzes, by Frank Conway. The publisher offers to send the lot post free on receipt of $1 00.

